# The pedunculopontine nucleus: From posture and locomotion to neuroepigenetics

**DOI:** 10.3934/Neuroscience.2019.4.219

**Published:** 2019-09-30

**Authors:** T. Virmani, F. J. Urbano, V. Bisagno, E. Garcia-Rill

**Affiliations:** 1 Center for Translational Neuroscience, University of Arkansas for Medical Sciences, Slot 847, Little Rock, AR 72205, USA; 2 Department of Neurology, University of Arkansas for Medical Sciences (UAMS), Little Rock, AR, USA; 3 Instituto Nacional de Investigaciones Farmacologicas, Argentina; 4 Universidad de Buenos Aires, Buenos Aires, Argentina

**Keywords:** arousal, deep brain stimulation, F-actin, histone deacetylase, N- and P/Q-type calcium channels, REM sleep, trichostatin A, waking

## Abstract

In this review, we discuss first an example of one of the symptoms of PD, freezing of gait (FOG), then we will turn to the use of deep brain stimulation (DBS) of the pedunculopontine nucleus (PPN) to treat PD, and the original studies that led to identification of the PPN as one source of locomotor control and why stimulation frequency is critical, and then describe the intrinsic properties of PPN neurons that require beta/gamma stimulation in order to fully activate all types of PPN neurons. Finally, we will describe recent findings on the proteomic and molecular consequences of gamma band activity in PPN neurons, with emphasis on the potential neuroepigenetic sequelae. These considerations will provide essential information for the appropriate refining and testing of PPN DBS as a potential therapy for PD, as well as alternative options.

## 1. Introduction

Deep brain stimulation (DBS) of the pedunculopontine nucleus (PPN) has been used for the treatment of Parkinson's disease (PD) with beneficial effects on a number of functions, including decreasing freezing of gait (FOG), decreasing falls, and lessening balance problems. The use of PPN DBS arose from our studies on animals using PPN stimulation to drive locomotion on a treadmill [1],[2], and was later proposed for modulating gait in PD [3]. In this review, we will discuss first an example of one of the symptoms of PD, FOG, then we will turn to the original studies that led to identification of the PPN as one source of locomotor control and why stimulation frequency is critical, and then describe the intrinsic properties of PPN neurons that require beta/gamma stimulation in order to optimally activate these neurons. Finally, we will describe recent findings on the proteomic and molecular consequences of gamma band activity in PPN neurons, with emphasis on the potential neuroepigenetic sequelae. These considerations will provide essential information for the appropriate refining and testing of PPN DBS as a potential therapy for PD, as well as alternative options.

## 2. Freezing of gait

The effects of PPN DBS have been reviewed recently [4], and will not be reiterated, except to emphasize its beneficial effects in decreasing FOG and falls [5]–[9]. Additional effects also have been reviewed recently [10], including improvements in daytime sleepiness and REM sleep, as well as enhanced cognitive functions [11]–[14], and increased glucose utilization in the frontal lobe [15]. But, why is FOG in PD important?

FOG is one of the more debilitating motor complications of PD [16], manifested by the feet “sticking to the ground” during active movement. FOG most commonly occurs on initiating gait (start-hesitation), and/or on turning [17]. FOG leads to increased instability and falls [18],[19], potential morbidity from falls [20], development of the fear of falling [21], and decreased quality of life [22]. FOG ranges from 7.1% in early disease [23], to 92% in our autopsy confirmed cohort [24]. Due to its episodic nature, and the fact that levodopa can also partially treat FOG, the signs may be masked until severe FOG develops and intervention is too late. There are also at least two broad categories of FOG, levodopa responsive FOG (or OFF-state FOG) in which the symptoms of freezing improve and can even resolve with increasing levodopa dose, and levodopa unresponsive FOG (or ON-state FOG), in which freezing is not improved with higher levodopa doses, and can even worsen in some individuals [25]. Why some patients develop earlier FOG and why some go on to develop ON-state FOG is still not understood and ongoing longitudinal gait studies in our lab hope to address some of these issues.

There have been a number of models proposed in regards to the development of episodes of gait freezing (for detailed review see [26]). Briefly, these have been summarized as a (i) Threshold model [27], (ii) Interference model [28], (iii) Cognitive model [29], and (iv) Decoupling model [30]. According to the Threshold model [27], when a highly coupled motor task such as gait is impaired as in PD, the threshold to achieve breakdown (i.e. the freezing episode) is reduced, which is supported by increased gait abnormalities during continuous gait in freezers compared to non-freezers [31],[32]. The Interference model suggests that freezing episodes are secondary to a breakdown in cross-talk between different basal ganglia circuit loops (oculomotor, sensorimotor, associative and limbic), which in the setting of decreased neural reserve in PD, leads to temporary inhibition of the PPN and thereby a freezing episode. The Cognitive model proposed that baseline global executive dysfunction in PD freezers, compounded by higher levels of impairment in freezers on tasks requiring set-shifting and conflict resolution leads to freezing episodes in situations requiring rapid decision making. The frontostriatal networks and the hyperdirect pathway are implicated in this model. This is supported by baseline executive and visuospatial deficits in FOG subjects [33],[34]. In our autopsy confirmed cohort, earlier onset of FOG was associated with earlier onset of postural instability, dyskinesias, sudden OFF states, hallucinations, and cognitive complaints [24]. Faster progression of FOG severity was also associated with earlier onset of hallucinations [24]. Lastly, the Decoupling model suggests that the motor programs that allow for coordinated gait get decoupled from their trigger at the time of initiation of the first step leading to the initiation freezing episode.

**Figure 1.**
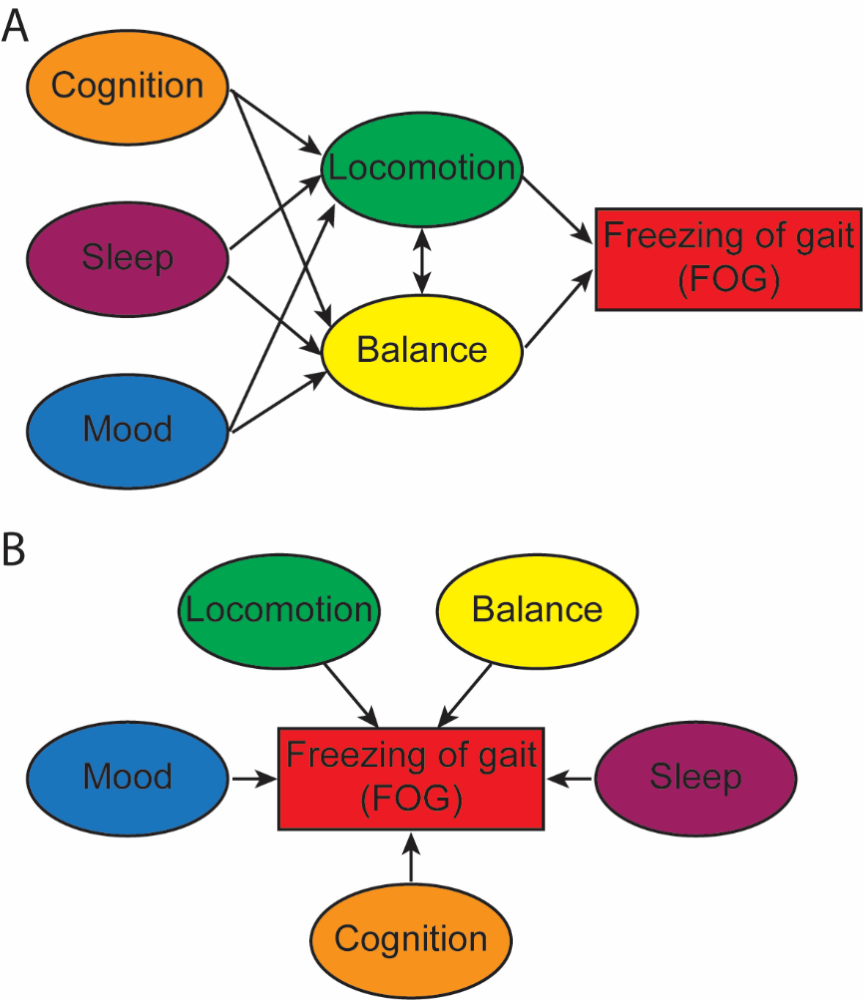
Examples of different network models for FOG. A. Sequential model with disruption of non-motor disease features such as cognition, sleep and mood, leading to impaired modulation of balance and locomotion circuitry, which then causes FOG. B. Multiple hit model with independent influence of different factors including gait and balance, possibly with different weight, leading to the eventual development of FOG once threshold for dysregulation is reached.

While all of these models have individual merit, when taken alone, none of them can adequately explain all aspects of freezing. Other than the Interference model, they do not take into account the parallel cognitive and limbic loops through the basal ganglia that also modulate movement. How the different loops interplay in the development of FOG is also unclear. One potential model integrating all aspects of gait control could be a Sequential model, as would be suggested based upon the Braak hypothesis of PD progression [35]. In this case for example, eventual cognitive involvement from Lewy body spread to the cortex could lead to breakdown in cognitive control of locomotion and balance circuitry, which on an already dysregulated system could lead to progressive freezing of gait (Figure 1A). Alternatively, a multiple hit model (Figure 1B) in which all the different components have some weight, and upon reaching that threshold of dysfunction, progressive FOG develops. The different levels of involvement of each of these components could lead to ON-state freezing of gait in either model. Large longitudinal cohorts of subjects, prospectively collecting multimodal information from PD subjects with and without freezing are needed to help shed light on these issues.

As the control of locomotion is currently best understood, we will focus on it for the remainder of this review.

## 3. Locomotor control

The PPN has always been recognized as a part of the reticular activating system (RAS), and thought to control waking and rapid eye movement (REM) sleep, two states of high frequency (beta/gamma) frequency in the cortical electroencephalogram (EEG) [36]. Stimulation of the region of the PPN led to the first descriptions of arousal following brain stem stimulation, including the induction of high frequency (beta/gamma) EEG activity [37]. Cholinergic cells in the PPN were found to project to the thalamus, and injection of cholinergic agonists into the thalamus was found to induce arousal and high frequency cortical EEG [38]. Cells in the PPN were reported to manifest activity in relation to waking (“Wake-on”), REM sleep (“REM on”), and both waking and REM sleep (“Wake-REM on”) [39]. However, we found that the same arousal-related region modulated locomotion and postural muscle tone.

The mesencephalic locomotor region (MLR) was an area of the posterior midbrain shown to induce locomotion on a treadmill in the decerebrate animal when stimulated at 40–60 Hz [40]. The region purportedly included the lateral cuneiform (but not medial cuneiform, nucleus, the posterior PPN, and portions of the ventral inferior colliculus and dorsal midbrain reticular formation. We determined that the PPN was an effective site that we could positively identify using immunocyto- and histo-chemical labeling [41],[42], while the other sites were not identifiable as distinct cell groups, although the activation of these regions with differing parameters could lead to stepping [2],[43]. We found that chemical stimulation of the PPN could be used to induce locomotion [44], suggesting that the consequences of electrical stimulation were not due to activation of fibers of passage; and we also found that single cell activity was present in the PPN in relation to locomotion [45], suggesting that the PPN contains cells active in relation to the start, cessation, and period of stepping. Moreover, descending projections of the PPN modulated regions related to postural muscle tone as well as regions driving spinal pattern generators responsible for stepping [2],[46],[47]. Further details of the determination of the role of the PPN are discussed in a recent review [4].

## 4. Three states, two channels

More recently, the mystery of why PPN stimulation requires 40–60 Hz frequency was resolved. The PPN is made up of cholinergic, glutamatergic and GABAergic cells [48]. We found that every PPN neuron, whether cholinergic, glutamatergic, or GABAergic, when stimulated fires maximally at gamma band frequency [49]. The reason for such a property is that PPN cells manifest high threshold, voltage-dependent calcium channels [50], and these channels subserve intrinsic beta/gamma oscillations that ensure that the natural frequency of PPN neurons is in the beta/gamma frequency [49],[50]. That is, PPN neurons have a preferred frequency of firing at 40–60 Hz. In addition, some cells (∼25%) manifested only N-type calcium channels, while others (∼25%) manifested only P/Q-type calcium channels, and most (∼50%) had both types of channels [51],[52]. The distribution of these channels was proposed to match up with *in vivo* firing patterns in the PPN showing “Wake-on” (firing only during waking, P/Q-type channels only), “REM-on” (firing only during REM sleep, N-type channels only), or “Wake-REM-on” (firing during waking and REM sleep, both N- and P/Q-type calcium channels) [51]–[54]. Therefore, the intrinsic properties of PPN neurons explain how this nucleus mediates waking and REM sleep.

Additionally, the two calcium channels involved are modulated by separate intracellular pathways, with calcium calmodulin kinase II (CaMKII) modulating P/Q-type channels and cyclic adenosine monophosphate/protein kinase (cAMP/PK) modulating N-type channels, both of which mediate intrinsic gamma oscillations [11]. Therefore, we proposed the presence of two channels with separate intracellular pathways control the states of waking vs REM sleep, as well as postural muscle tone and locomotion, as would be expected of an arousal-related region [4],[36],[55]. Below, we will address the intracellular mechanisms involved in the function of these channels, as well as the expression of proteins involved in the manifestation of intrinsic gamma oscillations.

## 5. Frequency matters

From the foregoing, it seems obvious that PPN neurons, all of them, manifest high threshold, voltage-dependent calcium channels, and those channels support beta/gamma frequency membrane oscillations. Unfortunately, a number of authors fail to appreciate the “natural frequency” that appears to activate PPN neurons. That is, by stimulating at such preferred frequencies as beta/gamma, the cells are more likely to fire and maintain firing at that preferred frequency. The converse is also true. By stimulating at frequencies outside the natural frequency of these cells, PPN neurons will not be activated or may indeed be inhibited, thus having opposite effects. For example, stimulating the PPN at high frequencies such as 100–300 Hz [56], or by sudden onset stimulation mimicking a startle response [57], PPN neurons will be depolarization blocked, inducing decreased postural muscle tone instead of locomotion. On the other hand, slowly ramping stimulation, whether stimulating the whole nucleus [4], or stimulating single cells [50], the depolarization will bring the membrane potential to its optimal firing frequency. It is surprising how few PPN DBS studies have actually tested 40–60 Hz consistently, with most beneficial effects observed at the low edge of this range (∼25 Hz) or above it (60–80 Hz), but not within the optimal range and, moreover, failing to appropriately test effects on sleep-wake and arousal regulation [58]. Thus, there are well established physiological reasons for stimulating the PPN at its preferred frequencies, but it seems that those designing the clinical parameters fail to appreciate the considerable of evidence demonstrating the optimal parameters required for PPN stimulation.

## 6. Histone deacetylation and F-actin

**Figure 2.**
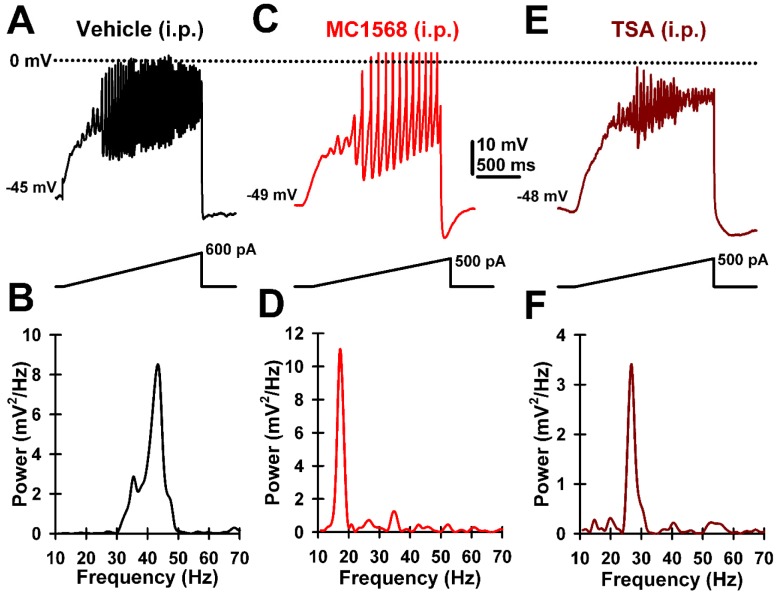
Effects of histone deacetylation inhibitors on PPN gamma oscillations. A. Ramp-induced intrinsic membrane oscillations in a PPN neuron. B. Ramp-induced oscillations 10 min after exposure to MC1568 are decreased in frequency. C. Ramp-induced oscillations 10 min after exposure to TSA are decreased in amplitude and frequency. D. Power spectrum of typical gamma oscillations induced in a PPN neuron, note frequency in the 40+ Hz range. E. Power spectrum of gamma oscillations after MC1568, note decrease in frequency to <10 Hz. F. Power spectrum of gamma oscillations in a PPN neuron after exposure to TSA, note reduction in amplitude and frequency to the beta range.

We recently showed that the manifestation of intrinsic gamma oscillations in PPN neurons are modulated by histone deacetylation (HDAC). Histone post-translational modifications, along with DNA demethylation, regulate gene expression in response to environmental stimuli, so-called neuroepigenetic regulation. We used trichostatin A (TSA) to block high threshold calcium channel-mediated oscillations, specifically in the gamma range and not lower frequencies, and particularly in cells manifesting P/Q-type channels [59]. That is, histone deacetylation inhibition specifically modulated oscillations related to waking. Figure 2 summarizes these results, showing that ramp-induced membrane oscillations in PPN neurons typically in the gamma range (Figure 2A and 2B), were reduced by the HDAC IIa inhibitor MC1568 (Figure 2C and 2D), and by the HDAC I and HDAC II inhibitor TSA (Figure 2E and 2F). These findings suggested that gamma oscillations in PPN neurons with P/Q-type channels, which are supported by CaMKII, are modulated by HDAC IIa [59]. Moreover, we showed that TSA administered in vivo had the same effects in blocking PPN gamma oscillations as when administered in vitro in slices [60]. TSA was found to block gamma oscillations in slices stimulated chemically using the cholinergic agonist carbachol, as well as when the PPN was stimulated electrically at 40 Hz [61]. But, do these effects change the expression of proteins in the PPN?

**Figure 3.**
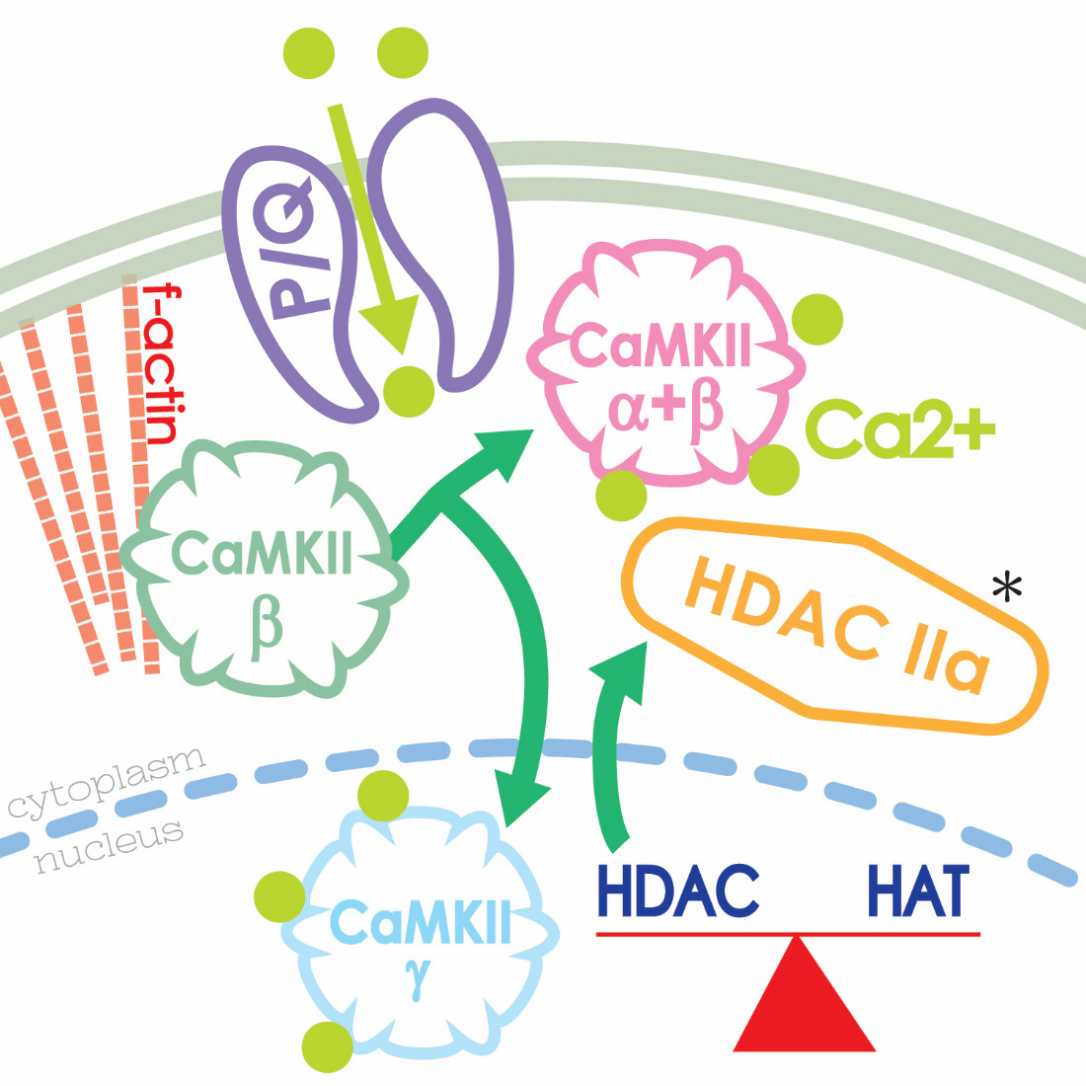
Intracellular mechanisms subserving proteomic events during gamma oscillations. High threshold, voltage-dependent P/Q-type calcium channels (purple) involved in gamma oscillations during waking interact with CaMKII (light green) and bind calcium (pink). F-actin (salmon) polymerizes to organize the interaction between CaMKII and HDAC IIa (yellow) which leaves to nucleus to trigger protein synthesis. The HDAC-HAT balance regulates protein synthesis and is modulated by CaMKII (blue) in the nucleus.

A proteomic study using a novel method of sampling only the PPN after exposure to carbachol or following 40 Hz electrical stimulation, revealed that the proteins normally regulated by this process were related to intracellular calcium regulation, such as calcineurin and neuronal calcium sensor 1 protein, as well as structural proteins such as F-actin [62]. Knowing that certain specific proteins are modulated by histone deacetylation inhibition, it remained to determine the mechanistic link between P/Q-type calcium channels, CaMKII, and HDAC IIa. Finally, we investigated the role of F-actin in the modulation of gamma oscillations in the PPN. We used agents that affect F-actin polymerization and discovered that a promoter of F-actin stabilization blocked PPN gamma oscillations through CaMKII (since a blocker of CaMKII prevented the effects of F-actin agents [63]. In summary, our results suggest that the mechanism between the influx of calcium through wake-promoting P/Q-type calcium channels and CaMKII, and the effects of HDAC IIa is related to F-actin. Figure 3 summarizes the proposed interactions between P/Q-type channels, CaMKII, and F-actin in the manifestation of gamma oscillations in PPN neurons.

## 7. Future directions

Firm conclusions can be drawn from knowledge of the physiology of the PPN. (1) Stimulation hoping to activate PPN neurons needs to be at 40–60 Hz. (2) Manipulation of PPN activity can be effected using agents that modulate CaMKII action to promote waking gamma band activity through P/Q-type channels, or cAMP/PK action to promote REM sleep gamma activity through N-type channels. (3) Another discovery not addressed here but described elsewhere [63] is that RAS centers manifest electrical coupling and modafinil, an atypical stimulant, acts by increasing electrical coupling. This decreases GABA release and increases other transmitters, promoting higher coherence at low frequencies (more smooth sleep) and high frequencies (promoting waking) [64]. All of these avenues need to be explored for the treatment of PD.
